# A Protein Corona‐Based Diagnostic Tool for Eroded Atherosclerotic Plaques

**DOI:** 10.1002/smll.202503915

**Published:** 2025-05-05

**Authors:** Santiago Alonso Tobar Leitão, Grasiele Sausen, Yuan Liu, Yanbao Yu, Antonietta Greco, Roberto Molinaro, Omid C. Farokhzad, Claudia Corbo, Peter Libby

**Affiliations:** ^1^ Center for Excellence in Vascular Biology Brigham and Women's Hospital Harvard Medical School 77 Louis Pasteur Ave Boston MA 02115 USA; ^2^ Center for Nanomedicine and Department of Anesthesiology Brigham and Women's Hospital Harvard Medical School 75 Francis Street Boston MA 02115 USA; ^3^ Seer Inc. 3800 Bridge Pkwy Redwood City CA 94065 USA; ^4^ Department of Chemistry and Biochemistry University of Delaware Newark DE 19716 USA; ^5^ School of Medicine and Surgery Nanomedicine Center Nanomib University of Milano‐Bicocca Via R. Follereau 3 Vedano al Lambro MB 20854 Italy; ^6^ IRCCS Istituto Ortopedico Galeazzi Via Cristina Belgioioso 173 Milan 20161 Italy

**Keywords:** atherosclerosis, metal‐organic framework nanoparticles, nano‐based blood assay, protein corona, superficial plaque erosion

## Abstract

Superficial plaque erosion, distinct from plaque rupture, represents a cause of residual thrombotic complications in atherosclerosis. Being able to detect plaque erosion, a mechanism that now accounts for up to 30% of acute coronary syndromes, can lead to targeted therapies, and perhaps avoid the need for urgent invasive interventions required for plaque rupture cases, thus greatly affecting clinical management. Nanoparticles (NPs) exposed to biological fluids acquire a layer of proteins on their surface, the protein corona (PC). Variations in the composition of the plasma proteome occurring in pathological conditions directly influence the composition of the PC. An NP‐based diagnostic approach is presented exploiting the proteomic changes at the nano‐bio interface for superficial plaque erosion detection. To this aim, an in vivo experimental murine procedure is used that recapitulates features of the pathophysiological conditions of superficial erosion. Two types of metal‐organicframework (MOF) NPs for PC generation are employed: zirconium and iron (III) MOF (ZrMOF and FeMOF). It is demonstrated that Zr‐MOF NPs act as plasma protein concentrators allowing the identification of a higher number of proteins, compared to unfractionated analysis of plasma. Nonetheless, It is identified thirteen plasma proteins exclusive to the superficial erosion group that might serve as signature proteins to discriminate the pathological condition.

## Graphical abstract

Protein Corona (PC) is a layer of proteins formed on the surface of nanoparticles (NPs) that are used as a tool for discriminati onof an experimental superficial erosion atherosclerotic injury in mice. Through a selection of different metals in the composition of metal‐organic framework (MOF) NPs, distinct corona proteins are formed on its surface, as Zr‐MOF showed a higher capability to identify the atherosclerotic group.

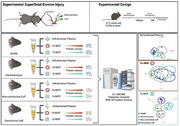

## 1 Introduction

Superficial plaque erosion has emerged as a growing cause of residual thrombotic complications of atherosclerosis, quite distinct from plaque rupture.^[^ ^1^ ^]^ In an era of improved prevention of traditional risk factors associated with acute coronary syndromes (ACS), plaque erosion may assume greater clinical importance especially because understanding its mechanisms may lead to management strategies for ACS due to erosion that may avoid urgent invasive therapy, as for the case of rupture.^[^ ^2^ ^]^ Thus, an effective diagnostic test for superficial erosion could have a powerful clinical impact for more appropriate management of ACS. Nanotechnology exploits distinct features of specific disease conditions to deliver selective therapeutics to the target tissue. Recently, we have employed lipid‐polymer hybrid nanoparticles (NPs) to deliver an inhibitor of the enzyme protein arginine deiminase (PAD)‐4 selectively to the collagen IV‐rich basement membrane, a subendothelial structure generally exposed in the presence of plaque erosion.^[^ ^3^ ^]^ PAD‐4 inhibitor was selected to prevent the neutrophil extracellular traps (NETs) formation, which we have implicated in superficial erosion.^[^ ^4^, ^5^ ^]^ When tested in vivo in an experimental setting recapitulating the characteristics of the human eroded lesion, these NPs reduced NET formation and preserved intimal integrity and endothelial barrier function.^[^ ^3^ ^]^

When exposed to biofluids, NPs absorb on their surface a protein layer, known as the protein corona (PC).^[^ ^6^, ^7^ ^]^ As the PC depends on the distinct features of NPs and biofluid composition,^[^ ^8^ ^]^ several cutting‐edge recent studies have employed a plethora of diverse NPs for differential interrogation of biological samples, including deep analysis of plasma proteins, for the research of biomarkers and/or molecular signature. Interestingly, PC‐based approaches have been used to discriminate between patients with Alzheimer's disease,^[^ ^9^, ^10^ ^]^ breast cancer,^[^ ^11^, ^12^ ^]^ prostate cancer^[^ ^12^ ^]^ and lung cancer^[^ ^13^ ^]^ and healthy donors. More recently, in a cohort study, we have demonstrated for the first time that this approach can aid the detection of coronary artery disease.^[^ ^14^ ^]^ Metal‐organic framework (MOF) materials have seen a variety of industrial applications including gas storage, separations, and catalysis due to their structural properties, such as the tunable pore channels, and the large surface areas.^[^ ^15^, ^16^, ^17^ ^]^ Indeed, the use of nanoscale MOFs in medicine has become a topic of considerable interest and their behavior in the physiological systems has been recently investigated.^[^ ^18^ ^]^

This study tested the hypothesis that the PC analysis of MOFs NPs can provide deep plasma proteome interrogation and can inform the development of a non‐invasive test for the diagnosis of ACS due to erosion in mice. Such an approach may direct a more personalized management strategy.

## 2 Results

### 2.1 MOF Nanoparticles Protein Corona

**Figure 1:**
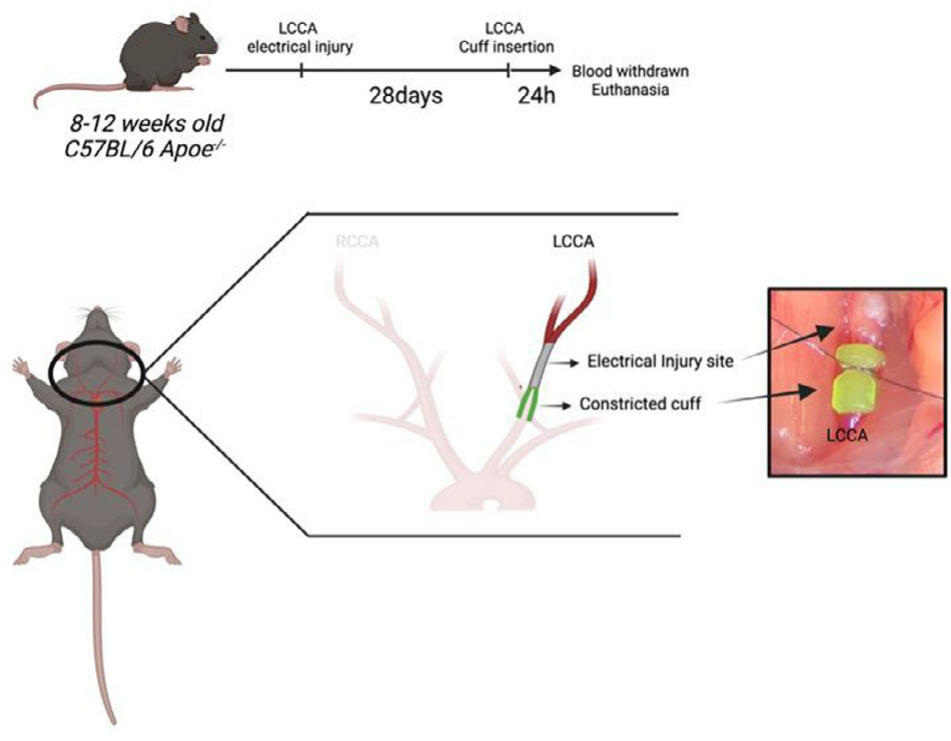
Study design. Eight to twelve weeks C57BL/6 APOE‐/‐ were submitted to an electrical injury in the left carotid artery (Sham controls). The lesion healed for 28 days before the placement of a constrictive cuff (non‐constricted cuff controls) just before the electrical injury site (picture). All animals were euthanized 24 h after cuff insertion.

For this study, we exposed mice to an experimental endothelial injury that recapitulates certain characteristics of superficial erosion in humans. Animal experimental design involves two sequential procedures; first, animals underwent a surgical artery electrical injury in the left carotid artery to produce an arterial intima that mimics important aspects of lesions that underlie thrombi due to erosion. The control of this procedure is a **Sham group, *n* = 7–10**. Then, 28 days after the electrical injury, a subset of animals that underwent carotid electrical injury (except **Electrical injury group**, ***n* = 7–12**) underwent a local flow disturbance elicited by placing a constricted cuff right before the electrical injury in the left carotid (**Cuff group, *n* = 11–12** recapitulating the superficial erosion). A further control group of mice had insertion of a non‐constrictive cuff placed in the same position (**Ncuff group**, ***n* = 8–10**). All animals were euthanized 24 h after the second surgery and their plasma was used for PC preparation. (**Figure** **1** and **Table** **1**).

**Table 1:** The sample size for each experimental group tested in the study.

		Experimental groups			
		SHAM	Electrical injury	NCuff	Cuff
Sample size	Unfractionated plasma	7	7	8	12
	Zr‐MOF	10	10	10	11
	Fe‐MOF	10	12	10	12

**Figure 2:**
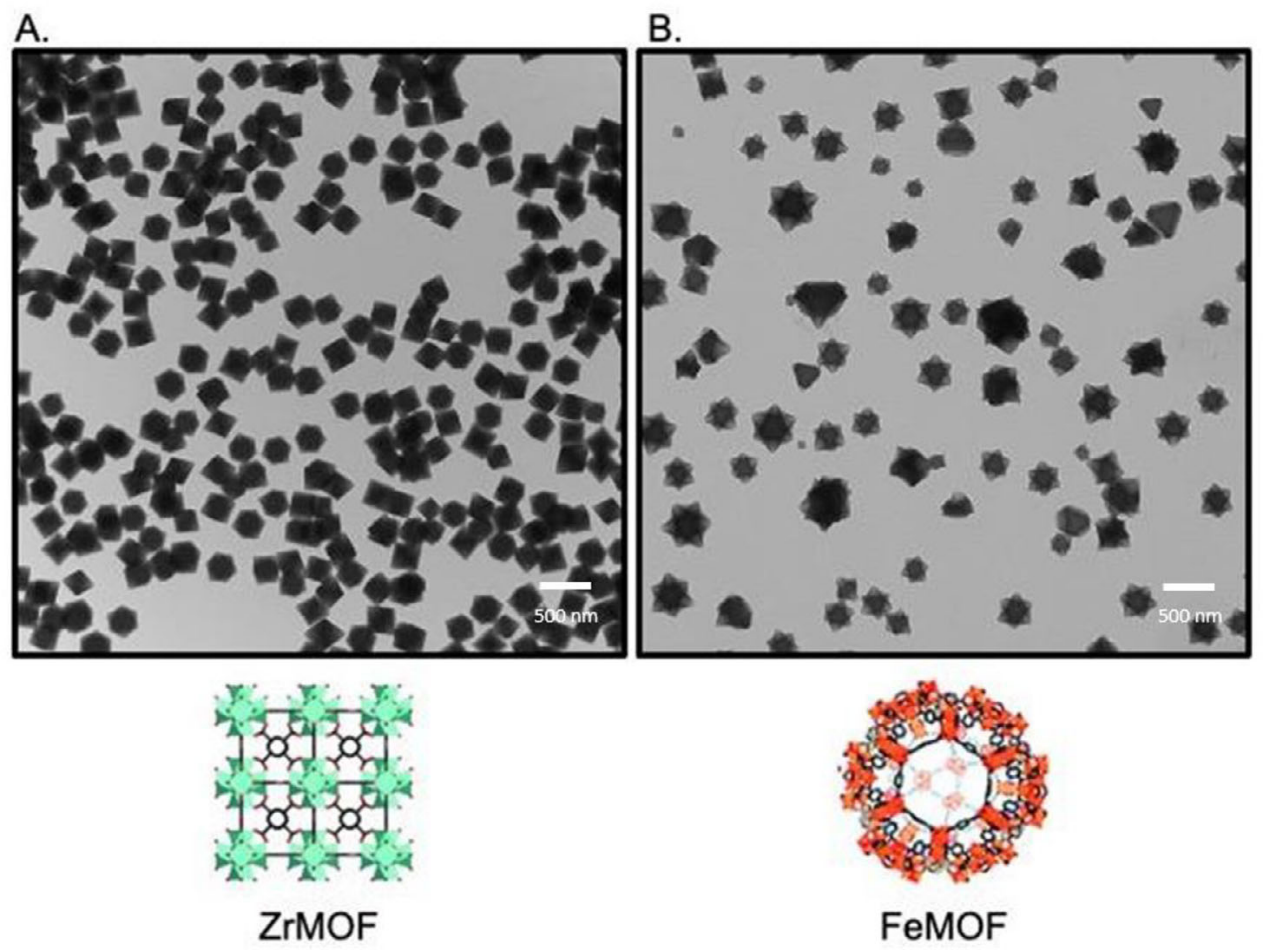
Transmission Electronic microscopy of MOF NPs. A. micrograph of ZrMOF and B. micrograph of FeMOF.

For the preparation of the PC, we used two distinct MOF NPs: Zirconium MOF (ZrMOF) and iron(III) MOF (FeMOF). The size of both the formulations was ≈300 nm with a uniform distribution (polydispersity index <0.3). NPs morphology was investigated by TEM analysis (*Section 4.3.3*), highlighting two different nano‐systems shapes: ZrMOF had a cubic structure (**Figure** **2**A) while FeMOF assumed a uniform octahedral morphology^[^ ^29^ ^]^ (Figure 2B).

**Figure 3:**
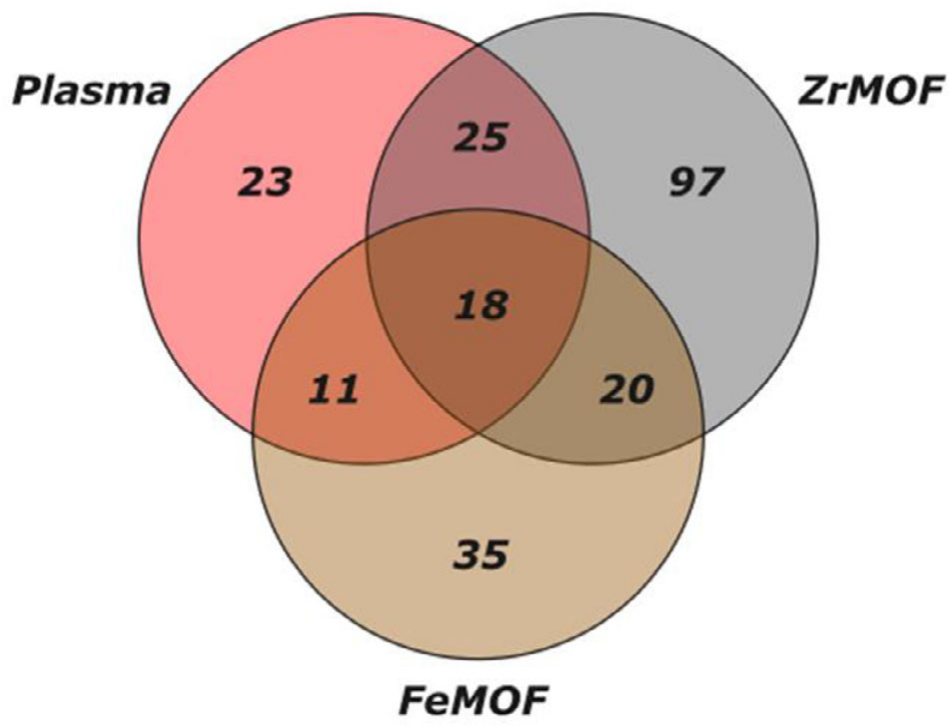
Venn Diagram of proteins extracted from ZrMOF or FeMOF, and plasma proteins. The annotated proteins extracted from each preparation are the differentially expressed proteins using ANOVA multiple group comparison (Sham, Electrical Injury, Non‐constricted Cuff, and Constricted Cuff) with a cut‐off in the FDR (*q* = 0.05) within each preparation.

To assess the NPs’ ability to interrogate plasma biomarkers for superficial erosion lesions in mice, 1 mg of MOF NPs were incubated with plasma of each group of mice to produce PC‐coated NPs. PCs were then processed for mass spectrometry (MS) based proteomics analysis following the suspension trapping (STrap) approach with in‐house packed filter devices. In parallel, to evaluate the actual advantage of using the PC, we also analyzed unfractionated plasma from each group. We detected 321 proteins in the corona of ZrMOF, 340 proteins in the corona of FeMOF, and 283 proteins in unfractionated plasma from mice in all 4 experimental groups (Supplementary Figure S1, Supporting Information). Then, we filtered these results to assess only the differentially expressed proteins across groups (Sham, Electrical Injury, NCuff, and Cuff) using multi‐group comparison with FDR < 0.05 at the Qlucore platform. This selection yielded 77 differentially expressed proteins for unfractionated plasma, 160 proteins in the PC of ZrMOF, and 84 proteins in the PC of FeMOF. Among all, only 18 proteins were common to all the data set, while 23 were identified exclusively in the unfractionated plasma dataset, 35 were exclusive of FeMOF's PC, and 97 were exclusive of ZrMOF's PC (**Figure** **3**).

**Figure 4:**
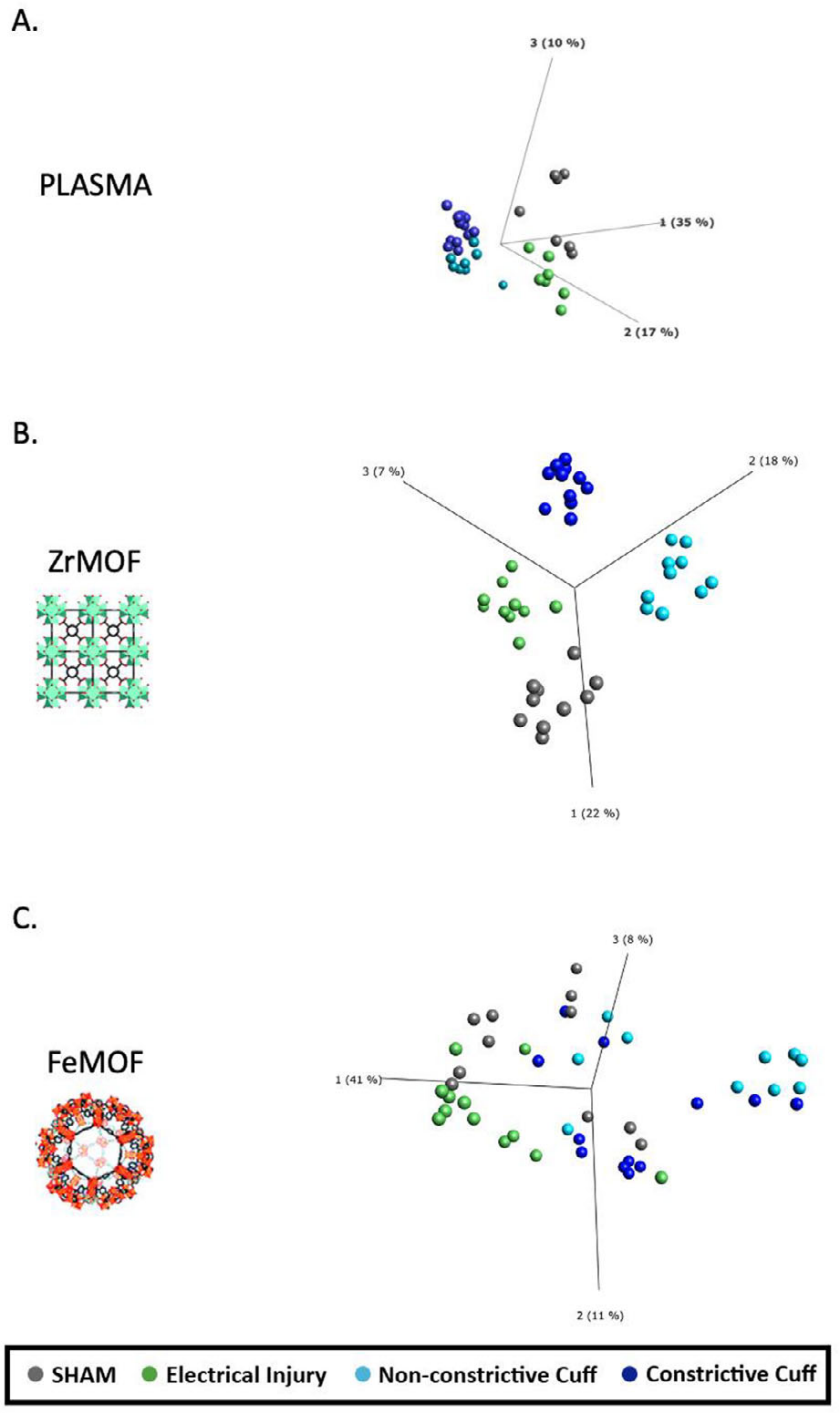
Principal Component Analysis (PCA). A) Plasma PCA; B) ZrMOF PC PCA; C) FeMOF PC PCA. Differentially expressed proteins were analyzed by multi‐group comparison with an FDR (q value) threshold of 0.05.

The differentially expressed proteins were then analyzed by the orthonormal basis of the principal component analysis (PCA) to assess the ability of each NP to differentiate the experimental groups. The differential proteomic analysis of unfractionated plasma allowed distinguishing Sham and Electrical injury animals (first surgery) from animals who underwent cuff placement (Ncuff and Cuff) (**Figure** **4**A), but did not differentiate animals with the experimental superficial erosion lesion (Cuff) from its control group (Ncuff) (Figure 4A).

More interestingly, our results demonstrated that the PCs of ZrMOF‐NP effectively differentiated all four groups (Figure 4B), while FeMOF did not discriminate among the groups (Figure 4C).

In light of these results, i.e., ZrMOF NPs performed best in the absorption of plasma proteins and ability to discriminate the different groups, we focused further on the ZrMOF‐NP PC to explore its content to determine the proteins that enabled the discrimination.

**Figure 5:**
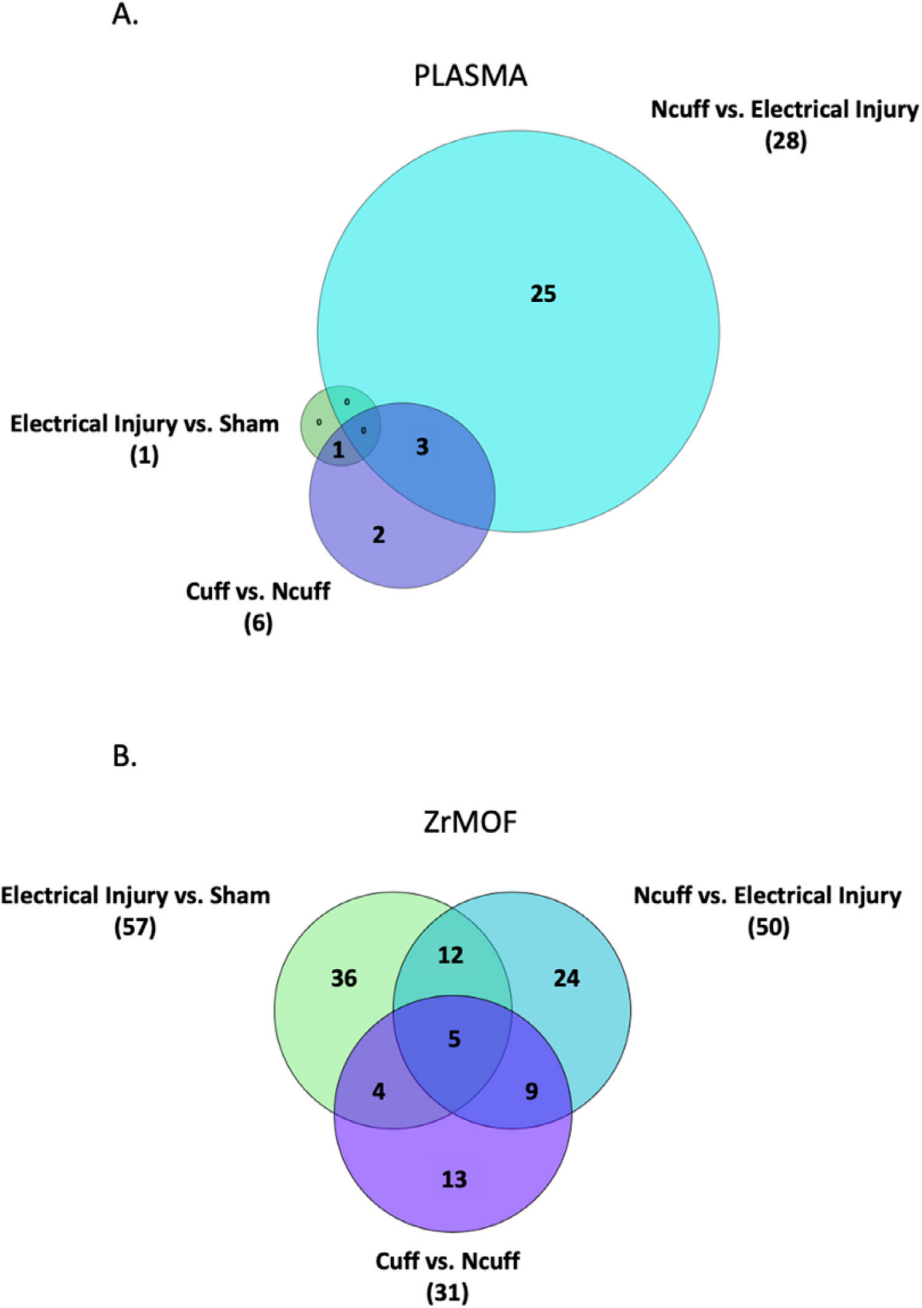
Venn diagrams of differentially expressed proteins in a two‐group comparison. A) pure plasma proteome analysis. B) PC‐based approach using ZrMOF for PC creation. In green are represented differentially represented proteins of the Electrical injury group versus Sham, in light blue Non‐constricted cuff group versus Electrical Injury group, and in dark blue Constrictive Cuff group versus Non‐constrictive cuff group.

To that end, we analyzed ZrMOF‐NP PC and plasma proteome using a two‐group comparison (FDR<0.05), as follows: i) Electrical Injury versus Sham group, ii) Ncuff versus Electrical Injury group, and iii) Cuff versus Ncuff group. The list of the differentially expressed proteins for each comparison was used to construct a Venn diagram. Differential analysis of unfractionated plasmas showed only one protein differentially represented in the Electrical Injury versus Sham group, while the PC of ZrMOF‐NP revealed 57 differentially represented proteins. In the comparison between the Electrical injury group versus Ncuff group, 28 proteins were differentially represented in unfractionated plasmas and 50 proteins in the PC of ZrMOF‐NP. Finally, in the comparison of Cuff versus Ncuff group, unfractionated plasma analysis revealed 6 proteins, while the PC of ZrMOF‐NP had 31 proteins suitable for discrimination (**Figure** **5**A,B, and Supplementary Figure S2, Supporting Information).

Interestingly, by overlapping common proteins in search for proteins exclusive for Cuff group, we found that the PC of Zr‐MOF‐NPs contained 13 unique proteins able to discriminate the superficial erosion from control, while only two unique proteins were identified in the traditional analysis of plasma proteome without the PC‐based approach (Figure 5A and B and Supplementary Table S1, Supporting Information).

**Figure 6:**
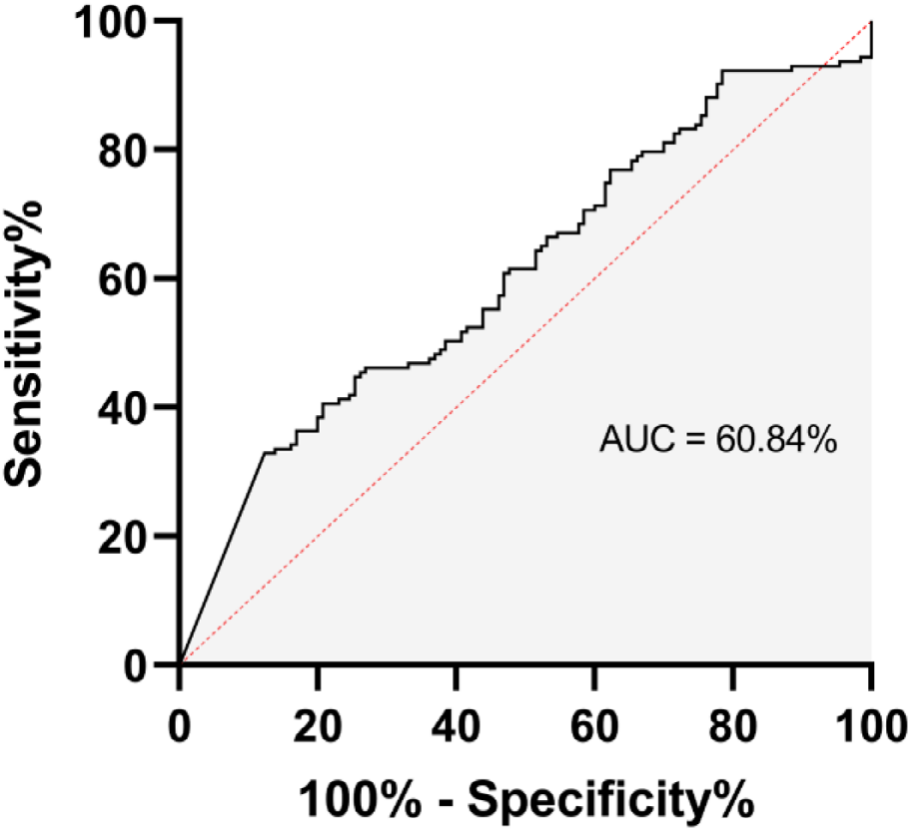
Area under the curve (AUC) observed by the analysis of the 13 unique proteins in the PC of Zr‐MOF‐NPs able to discriminate the superficial erosion from control (listed in Supplementary Table S1, Supporting Information).

Finally, to reinforce our findings, we tested the sensitivity and specificity of the differentially expressed proteins identified in the comparison between the groups constrictive cuff and non‐constrictive cuff, using the Receiver Operating Characteristics (ROC) curve. Analysis of the 13 proteins listed in Supplementary Table S1 (Supporting Information) yielded an area under the curve (AUC) of 60.84% was observed (**Figure** **6**). However, when these proteins were tested individually, the AUC ranged from 76.36% (MASP1) – 100% (HBB1) (**Table** **2** and Supplementary Figure S3, Supporting Information). A heatmap graph corresponding to the presence of the 13 differentially expressed proteins in the different experimental groups is also presented (Supplementary Figure S4, Supporting Information). Together, these results demonstrated the efficacy of the ZrMOF NPs in acting as a nano‐concentrator of proteins by means of PC formation.

**Table 2:** Area under the curve for each differential expressed protein of ZrMOF Nanoparticles in the comparison Cuff x Ncuff – Results from ROC analysis.

ZrMOF‐NP		
ENTRY	AUC (SEM)	*p* value
ALB	86.36 (8.4)	0.0049
CRYL1	81.82 (9.7)	0.0137
A2AP	89.09 (7.7)	0.0025
ADIPO	92.27 (6.6)	0.0011
CFAI	90.00 (6.9)	0.0019
GCAB	90.91 (8.7)	0.0015
HA10	80 (10.3)	0.0201
HBB1	100 (0.0)	0.0001
KV6A5	80.91 (10.12)	0.0167
LBP	99.09 (1.5)	0.0001
MASP1	76.36 (11.29)	0.0411
SPA3K	88.16 (9.0)	0.0031
VTDB	90.00 (7.2)	0.0019

## 3 Discussion

ACS remains the leading cause of morbidity and mortality worldwide. An evaluation of current culprit lesions suggests that superficial erosion has become more prominent as an ACS mechanism, accounting for up to a third of ACS, and may become a more prevalent cause of thrombotic complications in atherosclerosis in the era of highly effective lipid lowering.^[^ ^1^, ^19^ ^]^ Yet, the mechanisms of plaque erosion remain poorly understood; moreover, the diagnostic technologies to manage superficial erosion have received scant attention.^[^ ^20^ ^]^

Indeed, a non‐invasive strategy to accurately diagnose the plaques prone to disruption and stratify patients at risk is an urgent unmet medical need.^[^ ^21^, ^22^ ^]^ Many proposed methods involve invasive catheter‐based imaging techniques such as optical coherence tomography (OCT) that require major resources, consume time, and entail risk to patients. However, these techniques rely on morphological features rather than biological characteristics of lesions.^[^ ^20^, ^21^ ^]^ Despite its inability to identify endothelial lining^[^ ^20^ ^]^ OCT has served to identify culprit lesions that provoke ACS^[^ ^23^ ^]^ and suspect superficial erosion based on the integrity of the plaques fibrous cap to exclude plaque rupture. Nonetheless, despite the low risk of major complications (<1%), intravascular imaging may provoke serious complications, especially in older patients with comorbidities.^[^ ^24^ ^]^ In this setting, new technologies that could increase the accuracy for superficial erosion detection in a blood‐based test would prove very useful.

To address this problem, we created intimal lesions in mouse carotid arteries that resemble the substrate of human eroded plaques by having high extracellular matrix content, but scant macrophages or lipid accumulation. This preparation used electrical injury to the adventitia, followed by 4 weeks of healing to form a neointima and allow reendothelialization of the intimal surface. After this period of healing, we produced a flow perturbation by means of a periadventitial cuff. This intervention promoted downstream endothelial activation, neutrophil accumulation, death of endothelial cells, and their eventual desquamation.^[^ ^25^ ^]^

Using this approach, we employed a PC‐based method to point to the development of a diagnostic tool for superficial erosion. Indeed, in recent years, the PC of NPs has shown the capability to deeply interrogate the plasma proteome in search of biomarkers of a plethora of conditions. Our group and others have demonstrated that the creation of PCs on multiple NPs with different chemical and physical properties can help in increasing the accuracy of the discrimination power of the nanoplatform^[^ ^9^, ^13^ ^]^; for this reason, we evaluated the PC formation and composition in the two NPs platforms ZrMOF and FeMOF. It is important to highlight that the PC adsorption onto ZrMOF and FeMOF depends on the physicochemical properties of NPs, protein characteristics, and the biological environment. Moreover, well‐established factors such as NPs’ composition, size, surface charge, pH responsiveness, and morphology significantly influence PC formation.^[^ ^26^, ^27^, ^28^ ^]^ For example, the different crystal structure between ZrMOF and FeMOF can affect their affinity for proteins.^[^ ^29^, ^30^ ^]^ The same applies to the presence of different functional groups on the NP surface which enables interactions with specific biomolecules, facilitating the formation of hydrogen bonds and coordination with amino acid residues in proteins, a condition that stabilizes the corona.^[^ ^28^, ^31^ ^]^ Due to their tunable porosity and high surface area, MOF can adsorb large amounts of proteins or other biomolecules. Indeed, the extensive surface area provides more binding sites, increasing the probability of protein binding to the MOF's surface. Furthermore, the internal pores can entrap smaller biomolecules, contributing to the formation of the dynamic soft corona that can exchange with surrounding biomolecules in the environment.^[^ ^30^ ^]^ MOF serves to enhance the hydrogenation of α, and β‐unsaturated aldehydes, where the porous nature of the material leads to higher surface activity. However, the selectivity of MOFs for catalyzing α,β‐unsaturated aldehydes depends on the metal scaffold (Fe, Zr, Co, or Cr). Another interesting aspect which may impact the ZrMOF and FeMOF PC composition is that proteins, such as albumin, exhibit metal‐dependent (pseudo‐)enzymatic properties by contributing to plasma detoxification, pro‐drug activation, nucleic acid recognition, and the transport and storage of transition metals.^[^ ^31^ ^]^

We generated PCs by exposing two different MOF NPs, i.e., Zr and Fe, to the plasma from mice who underwent experimental superficial erosion injury or the plasma of various control animals. To assess the capability of our NPs to act as proteome concentrators, we also performed MS analysis also on the plasma proteome and compared the results. Our data showed that the PC formation on MOF NPs allowed us to identify a higher number of proteins, if compared to the analysis of plasma without PC formation (total number of identified proteins: Zr‐MOF – 321; Fe‐MOF – 340; and plasma – 283), thus affirming the capability of the PC to allow deep profiling of the plasma proteome.

The PC of Zr‐MOF revealed the highest number of differentially represented proteins within each group (Zr‐MOF – 160 proteins; Fe‐MOF – 83 proteins; and plasma – 77 proteins). Of note, in the PC of Zr‐MOF, 13 unique proteins differentiated the constricted cuff group (mimic of eroded plaque) from the non‐constricted cuff (control).

Our results provide proof‐of‐concept for the development of a blood‐based PC NP tool for the diagnosis of superficial erosion atherosclerotic lesions. The NPs concentrate those proteins showing the highest affinity toward their surface, thus allowing the detection not only of the highly abundant proteins but also of those that undergo slight changes in abundance in the presence of a disease. Furthermore, this approach was not based on the identification of specific, predetermined biomarkers allowing an unbiased interrogation of the unfractionated biofluid. In this study, one of the two MOF‐NPs employed allowed successful discrimination between samples. We envision that by choosing additional MOF‐NPs for PC creation, changing the MS acquisition method to better enhance identification of low abundance proteins and quantitation accuracy, and using other time points mirroring a less acute superficial erosion lesion, we could be able to expand the corona proteome, thus furtherly improving the discrimination performances.

Furthermore, our investigations used animal experiments and thus would require extension to humans, development of a biomarker signature distinct to erosion versus rupture, and external validation. Our approach could apply to several distinct ACS mechanisms such as calcified plaque, lipid rich lesions, and atheroma with abundant extracellular matrix, the substrate of superficial erosion. Taken together, our data paved the way for the use of the NPs’ PC as a non‐invasive approach for the development of a point‐of‐care test that could drastically alter the management of a substantial subset of patients with ACS.

## 4 Experimental Section

### Animals

All animal experiments were performed in accordance with the guidelines of the Animal Welfare Act and the Guide for the Care and Use of Laboratory Animals approved by the Institutional Animal Care and Use Committee of the Harvard Medical Area Standing Committee on Animals (Protocol #:2016N000293; PI: Libby, Peter). ApoE‐/‐ male mice (Jackson Laboratories, USA), 6–12 weeks of age, were housed in the Harvard Medical School Facilities at the New Research Building (Boston, MA, USA). ApoE‐/‐ mice consumed a normal chow diet with water ad libitum. Mice were certified free of common pathogens by the suppliers and were monitored by the Harvard Medical Area Standing Committee on Animals. Mice were anesthetized with 90–200 mg kg^−1^ ketamine/10 mg kg^−1^ xylazine intraperitoneally. After recovery from anesthesia, the animals consumed a standard chow diet and water ad libitum. Post‐operative analgesia was administered using 0.05–0.1 mg kg^−1^ of buprenorphine (first dose prior to animal recovery and second dose at 8–12 h from the first dose for the first day; once the second day). Post‐surgical animals were evaluated daily for a minimum period of 4 days as required by the BWH CCM institutional policy.

### Superficial Erosion Procedure

Arterial injury was performed according to.^[^ ^25^ ^]^ Briefly, Eight‐week‐old Apoe‐/‐ mice underwent electrical injury of the left common carotid artery (LCCA). The LCCA was exposed by an incision on the ventral side of the neck and injured by electric current using a bipolar microcoagulator (Erbe ICC 200, USA) delivered through the tips of microforceps positioned perpendicularly to the longitudinal axis of the artery. A uniform injury was induced by applying a current pulse two times every millimeter from beneath the LCCA bifurcation. The total injured distance encompassed ≈2 mm, depending on the anatomical variability of the artery. A current pulse of 3 W was delivered by the microcoagulator for 4 s each time. Twenty‐eight days later, a local flow perturbation was induced proximal to the healed injury, using constrictive polyethylene cuffs manufactured to our specifications by 3D‐stereolithography printing (Proto Labs, USA). The nonconstrictive proximal internal diameter (500 µm) of the cuff decreases gradually to become constrictive at its end (distal internal diameter: 250 µm). Previous work formally characterized the flow disturbance produced distal to the cuff using direct measurements and computational fluid dynamics. Animals that underwent a sham procedure (with no cuff placement) and those that had placement of a non‐constrictive device served as controls for the surgical manipulations and adventitial injury and inflammation.

Flow perturbation continued for 24 h and mice were euthanized by CO₂ inhalation, with a flow rate of 2.5 L min^−1^ for ≈5 min (≈3 min to consciousness loss plus ≈2 min after breathing stops). Then, whole blood was withdrawn through a heart puncture with a syringe rinsed with 0.5 M EDTA. Plasma samples were prepared by centrifugation at 18,000xg for 10 min at room temperature.

### Metalorganic Framework (MOF) Preparation—Synthesis of Iron Metal‐Organic Framework (FeMOF)

To synthesize the FeMOF NPs, Biphenyl‐4,4′‐dicarboxylic acid (57.5 mg, 0.346 mmol) and iron(III) chloride hexahydrate (93.4 mg, 0.346 mmol) were dissolved in 16 mL of N‐dimethylformamide (DMF). The resulting mixture solution was then heated to 160 °C quickly in an oil bath and incubated in the flask for 10 min at 160 °C. The reaction was stopped by cooling to room temperature and the resulting FeMOF NPs were collected by centrifugation at 15500x*g* for 10 min. The FeMOF was washed 3 times using DMF and finally redispersed in water for further use.^[^ ^29^ ^]^

### Metalorganic Framework (MOF) Preparation—Synthesis of Zirconium Metal‐Organic Framework (ZrMOF)

In a typical synthesis, Biphenyl‐4,4′‐dicarboxylic acid (100 mg) and zirconyl chloride octahydrate (21 mg) were dissolved in 4 mL of DMF, followed by adding 2 mL of acetic acid. The resulting solution was incubated in an oil bath at 120 °C for 12 h. ZrMOF NPs were washed with DMF 3 times and finally redispersed in water for further use.^[^ ^29^ ^]^

### Metalorganic Framework (MOF) Preparation—Transmission Electron Microscopy for MOF nanoparticles

Samples were adsorbed to the grid by floating the grid on a drop of sample for 1 min, liquid excess was removed with filter paper (Whatman #1) and examined in a JEOL 1200EX Transmission electron microscope or a TecnaiG^2^ Spirit BioTWIN, and images were recorded with an AMT 2k CCD camera.

### Protein Corona Preparation and Elution

MOF NPs (1 mg, 20 µL) were added to 200 µL of serum diluted in 50% of water. The resulting mixture was put on a shaking platform (800 rpm) and incubated for 1 h at room temperature to form a uniform NPs PC. Then, the NPs PC was washed with NaCl solution (300 mm) three times to get rid of the nonspecific and unstable soft PC.

To elute the hard PC from the NPs surface, 100 µL of 2% Sodium Dodecyl Sulphate (SDS) in water was added to the tube and transferred to a hot plate (95 °C) and incubated for 10 min to denature the PC and elute the protein. Then, the tube was centrifuged at 15500x*g* for 10 min to separate the eluted proteins from MOF NPs, 95 µL of supernatant was collected for further analysis.

### Mass Spectrometry Analysis—*Sample Preparation*

Protein corona eluted from the NPs were first mixed with 5% SDS and 20 mM Dithiothreitol (DTT), and heated at 95 °C for 10 min, then were subjected to STrap based digestion using in‐house packed filter devices and Whatman GF/F glass fiber membrane, as described previously.^[^ ^32^ ^]^ The digests were desalted using C18‐based StageTips (CDS Analytical LLC, Oxford, PA)^[^ ^33^ ^]^ dried in a SpeedVac (Savant SPD1030, Thermo Scientific), and stored under ‐80 °C until further analysis.

### Mass Spectrometry Analysis—Liquid Chromatography Mass Spectrometry Analysis

The LC–MS/MS analysis was carried out using an Ultimate 3000 nanoLC system coupled to a FLEX source and a Q Exactive mass spectrometer (Thermo Scientific) as previously described.^[^ ^34^ ^]^ Peptides were resuspended in LC buffer A (0.1% formic acid in water), and loaded onto a trap column (PepMap C18, 2 cm × 100 µm; Thermo Scientific) and then separated on an in‐house packed analytical column (C18 ReproSil, 3.0 µm, Dr. Maisch GmbH; 19 cm × 75 µm I.D.). The 220‐min LC gradient included: 2–35% buffer B (0.1% formic acid in acetonitrile) over 180 min; 35–80% buffer B over 10 min; back to 2% B in 5 min for equilibration after staying on 80% B for 5 min. MS data were acquired with a scan range of 350–1700 Da using the data‐dependent top‐10 method. The maximum injection time of 20 ms, and the AGC target of 1e6. MS/MS was performed via higher energy collisional dissociation (HCD) fragmentation with a target value of 5e5 and a maximum injection time of 100 ms. Full MS and MS/MS scans were acquired at a resolution of 70000 and 17500, respectively. Dynamic exclusion was set to 20 s.

### Mass Spectrometry Analysis—Protein Identification and Quantification

Protein identification and quantitation were performed using the MaxQuant‐Andromeda software suite (version 1.6.3.4) with most of the default settings,^[^ ^35^ ^]^ including 10 and 20 ppm mass tolerances for precursor and fragments, respectively; trypsin as enzyme with two missed cleavage sites; protein N‐terminal acetylation and methionine oxidation as variable modifications; cysteine carbamidomethylation as a fixed modification; peptide length with at least 7 amino acids. False discovery rate (FDR) was set at 1% for both proteins and peptides. A mouse database (17038 sequences; Reviewed only; version March 2019) downloaded from UniProt Knowledgebase (https://www.uniprot.org/) was used for the database search. The label‐free quantitation (LFQ) function was enabled for proteome‐wide quantitation.

The MaxQuant output dataset was deposited at Harvard Dataverse (https://doi.org/10.7910/DVN/XGOTLD).

### Mass Spectrometry Analysis—Computational and Statistical Analysis

Differentially expressed proteins were calculated between multiple groups as an averaged‐log_2_ normalized protein LFQ (intensities) with statistical filtering using a FDR (q‐value) of <0.05. Then, protein abundance trends (heatmaps with hierarchical clustering, PCA, bi‐dimensional uniform manifold approximation, and projection (2D‐umap) were analyzed.

For the construction of the Venn Diagram, two‐group comparison was performed with statistical filtering using a FDR (q‐value) of <0.05. The list of differentially expressed proteins between each comparison was then used for constructing the Venn diagram and the volcano plots. Finally, a heatmap and ROC curve were generated to analyze the sensitivity (%) versus specificity (%) of differentially expressed proteins reported in Supplementary Figure S4 (Supporting Information) and Supplementary Table S1 (Supporting Information) (Ncuff versus Cuff groups) using the GraphPad Prism 8.3.0.

All proteomics data analysis and figures were performed using Qlucore v3.4 (Qlucore AB, Lund, Sweden).

## Conflict of Interest

OCF has financial interest in PrognomiQ and Seer.

## Supporting information



Supporting Information

## Data Availability

The data that support the findings of this study are available from the corresponding author upon reasonable request.
