# Supplementary material for: A Protein Corona‐Based Diagnostic Tool for Eroded Atherosclerotic Plaques
Source: Small. 2025 May 5;21(31):2503915. doi: 10.1002/smll.202503915 (PMC12332804; doi:10.1002/smll.202503915)
Supplement: Supplementary file 1 — Supporting Information [file SMLL-21-2503915-s001.docx]

**Supplementary Material**

**A protein corona-based diagnostic tool for eroded atherosclerotic plaques.**

*Santiago Alonso Tobar Leitão^#^, Grasiele Sausen^#^, Yuan Liu, Yanbao Yu, Antonietta Greco, Roberto Molinaro, Omid C. Farokhzad, Claudia Corbo* and Peter Libby**

**Supplemetary Figures and Table legend**

**Supplementary Figure S1.** Venn Diagram of 321 proteins extracted from the corona of ZrMOF, 340 proteins extracted from the hard corona of FeMOF, and 283 proteins present in the unfractionated plasma from mice in all 4 experimental groups.

**Supplementary Figure S2.** Volcano plot diagram depicting the comparison of Cuff vs. Ncuff group.

**Supplementary Figure S3.** ROC Curves of the individual proteins studied.

**Supplementary Figure S4.** Heat maps of 13 differentially expressed proteins in the comparison of Cuff vs Ncuff.

**Supplementary Table S1.** Differentially expressed proteins exclusive in the constrictive Cuff group. Functions for each protein were extracted from Uniprot.org.


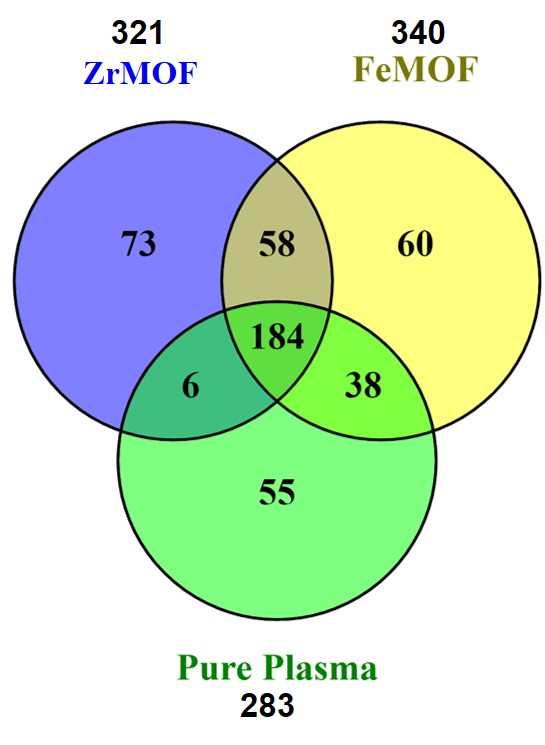


**Suppl. Figure S1**


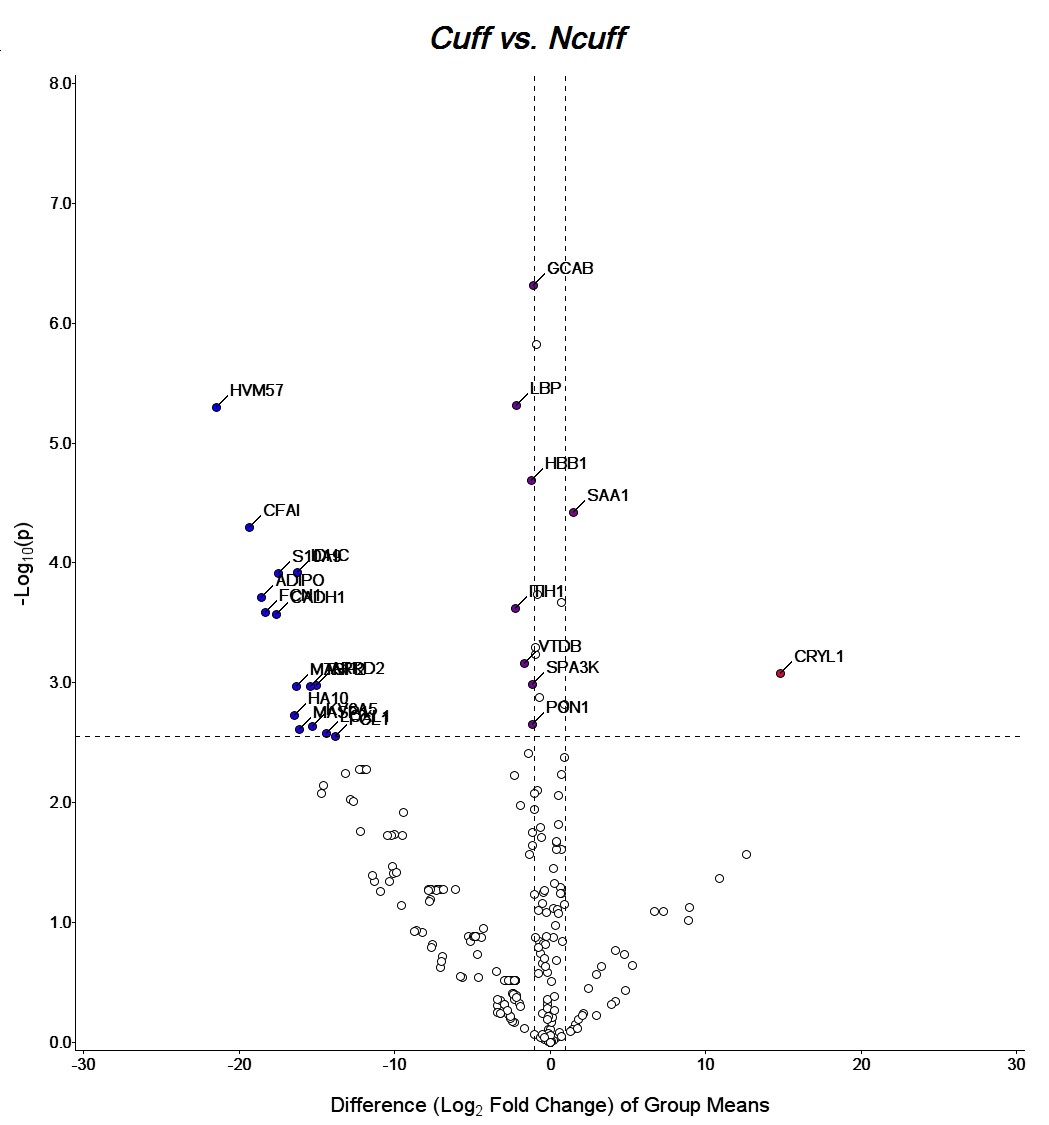


**Suppl. Figure S2**

**
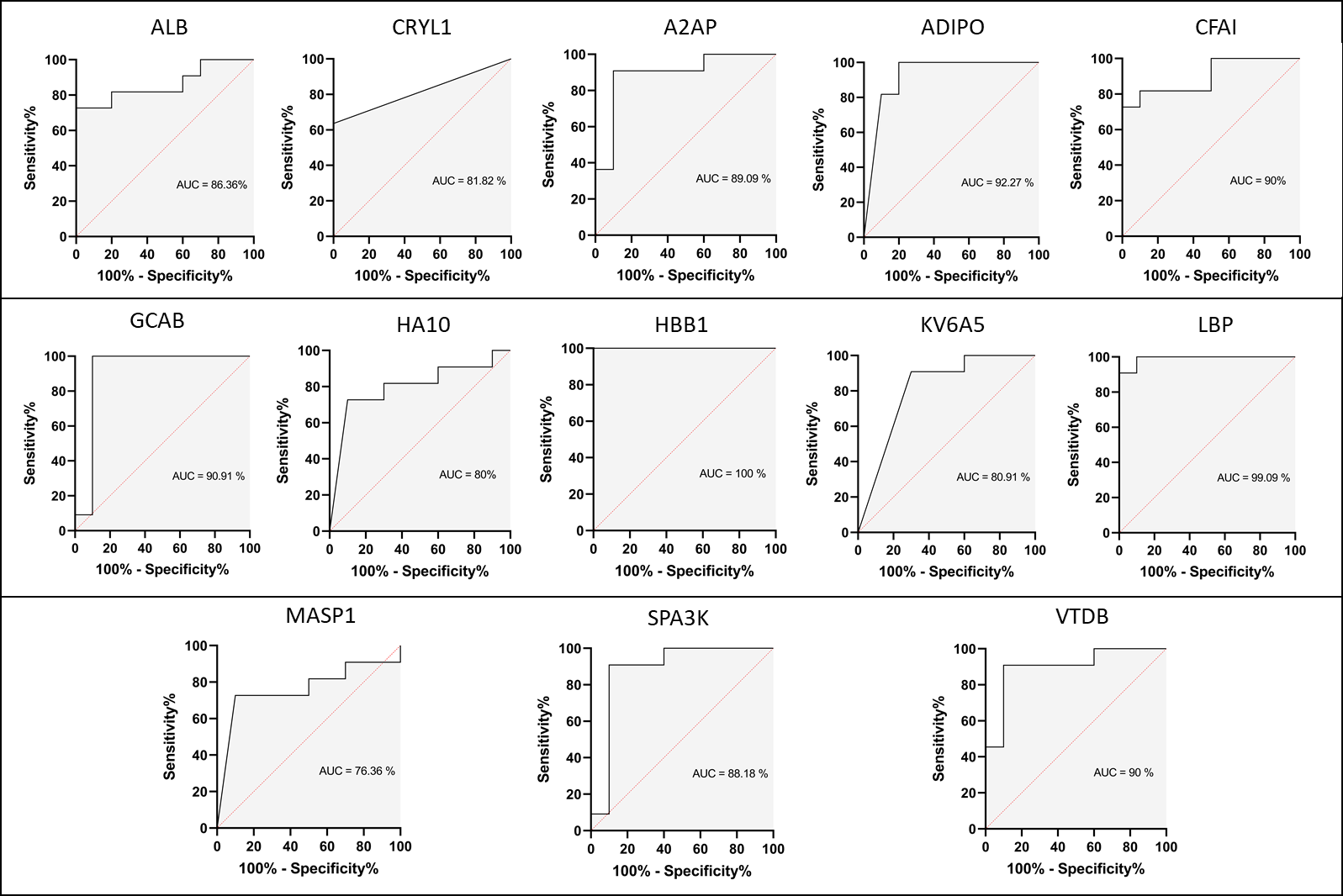
**

**Suppl. Figure S3**


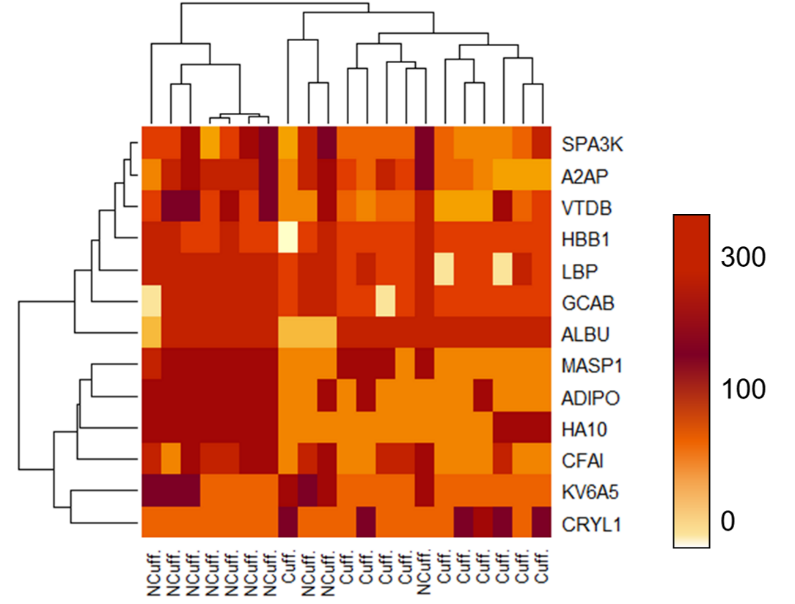


**Suppl. Figure S4**

| **Suppl. Table S1. Differentially expressed proteins exclusive in the constrictive Cuff group. Functions for each protein were extracted from Uniprot.org.** | | |
| --- | --- | --- |
| **ZrMOF-NP** | | |
| ENTRY | PROTEIN NAME | FUNCTION |
| A2AP | Alpha-2-antiplasmin | Alpha-2-antiplasmin; Serine protease inhibitor. The major targets of this inhibitor are plasmin and trypsin, but it also inactivates matriptase- 3/TMPRSS7 and chymotrypsin. (491 aa) |
| ADIPO | Adiponectin | Adiponectin; Important adipokine involved in the control of fat metabolism and insulin sensitivity, with direct anti-diabetic, anti-atherogenic, and anti-inflammatory activities. Stimulates AMPK phosphorylation and activation in the liver and the skeletal muscle, enhancing glucose utilization and fatty-acid combustion. Antagonizes TNF-alpha by negatively regulating its expression in various tissues such as liver and macrophages, and also by counteracting its effects. Inhibits endothelial NF-kappa-B signaling through a cAMP-dependent pathway. May play a role in cell growth, angiogenesis, and tissue remodeling by binding and sequestering various growth factors with distinct binding affinities, depending on the type of complex, LMW, MMW, or HMW. (244 aa) |
| ALBU | Serum albumin | Serum albumin, the main protein of plasma, has a good binding capacity for water, Ca(2+), Na(+), K(+), fatty acids, hormones, bilirubin, and drugs (Probable). Its main function is the regulation of the colloidal osmotic pressure of blood (Probable). Major zinc transporter in plasma typically binds about 80% of all plasma zinc. Major calcium and magnesium transporter in plasma binds approximately 45% of circulating calcium and magnesium in plasma (By similarity). Potentially has more than two calcium-binding sites and might additionally bind calcium in a non-specific manner (By similarity). The shared binding site between zinc and calcium at residue Asp-273 suggests a crosstalk between zinc and calcium transport in the blood (By similarity). (609 aa) |
| CFAI | Complement factor I | Complement factor I heavy chain; Trypsin-like serine protease that plays an essential role in regulating the immune response by controlling all complement pathways. Inhibits these pathways by cleaving three peptide bonds in the alpha-chain of C3b and two bonds in the alpha-chain of C4b thereby inactivating these proteins. Essential cofactors for these reactions include factor H and C4BP in the fluid phase and membrane cofactor protein/CD46 and CR1 on cell surfaces.  The presence of these cofactors in healthy cells allows degradation of deposited C3b by CFI to prevent undesired complement activation, while in apoptotic cells or microbes, the absence of such cofactors leads to C3b-mediated complement activation and subsequent opsonization. (591 aa) |
| CRYL1 | Lambda-crystallin homolog | Lambda-crystallin homolog; Crystallin lambda 1. (319 aa) |
| GCAB | Ig gamma-2A chain C region secreted form |  |
| HA10 | H-2 class I histocompatibility antigen, Q10 alpha chain |  |
| HBB1 | Hemoglobin subunit beta-1 |  |
| KV6A5 | Ig kappa chain V-VI region J539 |  |
| LBP | Lipopolysaccharide-binding protein | Lipopolysaccharide-binding protein; Plays a role in the innate immune response. Binds to the lipid A moiety of bacterial lipopolysaccharides (LPS), a glycolipid present in the outer membrane of all Gram-negative bacteria. Acts as an affinity enhancer for CD14, facilitating its association with LPS. Promotes the release of cytokines in response to bacterial lipopolysaccharide. (481 aa) |
| MASP1 | Mannan-binding lectin serine protease 1 | Mannan binding lectin serine peptidase 1. (728 aa) |
| SPA3K | Serine protease inhibitor A3K | Alpha-2-antiplasmin; Serine protease inhibitor. The major targets of this inhibitor are plasmin and trypsin, but it also inactivates matriptase- 3/TMPRSS7 and chymotrypsin. (491 aa) |
| VTDB | Vitamin D-binding protein | Vitamin D-binding protein; Involved in vitamin D transport and storage, scavenging of extracellular G-actin, enhancement of the chemotactic activity of C5 alpha for neutrophils in inflammation and macrophage activation. Belongs to the ALB/AFP/VDB family. (493 aa) |
